# Knowledge and attitudes of doctors regarding the provision of mental health care in Doddaballapur Taluk, Bangalore Rural district, Karnataka

**DOI:** 10.1186/1752-4458-6-21

**Published:** 2012-09-21

**Authors:** Joshua Cowan, Shoba Raja, Amali Naik, Gregory Armstrong

**Affiliations:** 1 Nossal Institute for Global Health, University of Melbourne, Melbourne, Australia; 2 Basic Needs, Bangalore, India; 3 Gramina Abrudaya Seva Samastha, Doddaballapur, Bangalore, India

## Abstract

**Background:**

Specialist mental health care is out of reach for most Indians. The World Health Organisation has called for the integration of mental health into primary health care as a key strategy in closing the treatment gap. However, few studies in India have examined medical practitioners’ mental health-related knowledge and attitudes. This study examined these facets of service provision amongst doctors providing primary health care in a rural area of Karnataka is Southern India.

**Methods:**

A mental health knowledge and attitudes questionnaire was self- administered by participants. The questionnaire consisted of four sections; 1) basic demographics and practice information, 2) training in mental health, 3) knowledge of mental health, and self-perceived competence in providing mental health care, and 4) attitudes towards mental health. Data was analysed quantitatively, primarily using descriptive statistics.

**Results:**

This study recruited 46 participants. The majority of participants (69.6%) felt competent in providing mental health services to their patients. However, there was a substantial level of endorsement for several statements that reflected negative attitudes. Almost one third of participants (28.0%) had not received any training in providing mental health care. Whilst three-quarters of participants correctly identified depression (76.1%) and psychosis (76.1%) in a vignette, fewer were able to name three common signs and symptoms of depression (50.0%) and psychosis (28.3%).

**Conclusions:**

Integrating mental health into primary health care requires evidence-based up-skilling programs. Doctors in this study desired such training and would benefit from it, with a focus on both depth of knowledge and uncovering stigmatising attitudes towards people with mental health problems.

## Background

Mental disorders are estimated to account for 14% of all disability adjusted life years (DALY’s) lost to disease worldwide [1,2], and contribute more to the global burden of disease than either cardiovascular disease or cancer [1]. Mental disorders are associated with a range of other communicable and non-communicable health problems, underpinning the catchphrase “no health without mental health” [1,3].

There are vast shortages in the available supply of human resources for providing mental health care in most low and middle-income countries, and these shortages are likely to persist [4,5]. For example, in lower-middle income countries the average number of psychiatrists per 100,000 population is 1.1, compared to 10.0 in high income countries [6]. The contrast in access to mental health specialists between lower-middle and high income countries is even greater when we compare the disparity in the numbers of psychologists, psychiatric nurses, and mental health social workers.

In India, approximately 6% of the population have a mental disorder [3], and suicide is a major public health problem [7]. The vast majority of care is provided by the family, and the understanding and experience of mental disorders is not necessarily the same as it is in Western countries [8-12]. Many people do not receive mental health care and will often turn to non-allopathic providers such as traditional, indigenous, religious, and alternative healers [10,11,13]. Specialist mental health care is out of the reach of most Indians living with a mental disorder due to the scarcity of mental health professionals and the relatively high cost of accessing good quality care in the private market [3,7,14].

The World Health Organization (WHO) has called for the integration of mental health into primary health care (PHC) as a key strategy in closing the treatment gap [15]. Part of this strategy requires a level of task shifting so that mental health care is provided by a range of PHC personnel and not just specialists such as psychiatrists and psychologists [16]. The kinds of health personnel in low-income settings that may be able to play a key role in providing basic mental health care include, for example, PHC doctors and nurses, village health workers and community health workers.

Currently India has no national mental health policy, but it does have a National Mental Health Programme (NMHP) that functions in its place [11]. The NMHP is also advocating the need to integrate mental health into PHC. However, there has been limited success in realising this policy in practice with only 125 of 626 districts currently covered by this program [3,6,7,17].

People with mental disorders frequently present to PHC settings due to the overlap between physical health and mental health and the somatisation of distress [18,19]. One study in a Mumbai slum, for example, found that 28% of patients aged > 18 years suffered from psychiatric problems [9]. However, PHC personnel require sufficient knowledge, skills and attitudes in order to competently and effectively provide a reasonable standard of mental health care. Yet few studies in India have examined medical practitioners’ mental health-related knowledge and attitudes [20].

Only three relatively recent studies (two urban and one rural) investigating mental health-related knowledge, attitudes and practices of PHC doctors in India were identified [20-22]. The findings typically indicated a lack of knowledge of diagnostic criteria, insufficient training in mental health, and a mixed range of positive and negative attitudes regarding mental health. A small number of studies have been examining various aspects of integrating mental health into PHC in India [23-27]. This is creating momentum for achieving real changes in practice, with the aim of seeking better outcomes for people with mental disorders. This study makes a contribution to this body of evidence by examining the mental health-related knowledge and attitudes of doctors providing PHC in a rural area of Karnataka in Southern India. The information generated will be important for the further development of appropriate up-skilling tools and resources.

## Methods

We administered a mental health knowledge and attitudes questionnaire to doctors providing PHC in Doddaballapur Taluk, Rural Bangalore District, Karnataka, India. The research was approved by The University of Melbourne’s Human Research Ethics Committee. Data for this project was collected during January and February 2011.

### Participants and sample size

The study participants were both government and private MBBS-trained doctors operating in Doddaballapur Taluk. Ayurvedic and homeopathic-trained doctors operating in the government sector were also included as participants given that they were providing primary care in partnership with MBBS-trained doctors and were practicing under the same government regulations. The doctors were identified through the networks of Gramina Abrudaya Seva Samstha (GASS), an NGO operating in Doddaballapur. There were 72 doctors (50 private and 22 public; 68 MBBS and 4 ayurvedic/homeopathic) operating in Doddaballapur who were eligible to participate in the study. Government MBBS-trained doctors were general practitioners. Some of the private doctors had further qualifications in such fields as obstetrics and gynaecology, paediatrics, and surgery, and were included in the sample as they were providing primary medical care and seeing general “walk-ins”. None were specialists in psychiatry.

### Questionnaire Design

The questionnaire was self-administered and consisted of four sections; 1) basic demographics and practice information, 2) training in mental health, 3) knowledge of mental health, and self-perceived competence in providing mental health care, and 4) attitudes towards mental health.

To assess knowledge of mental health, participants were read two vignettes describing people experiencing symptoms of mental disorders (one depression and the other psychosis) and subsequently asked to identify the problems. The vignettes (see below) were taken from a mental health literacy questionnaire previously used in India and elsewhere [28-31]. Additionally participants were asked to name three common signs or symptoms of both depression and psychosis; responses to these questions were pre-identified as correct or incorrect in consultation with a culturally appropriate psychiatry text [32]. Attitudes towards mental health were examined by asking participants to agree or disagree with a series of attitudinal statements adapted from the mental health literacy questionnaire and a depression attitudes questionnaire previously used with primary care practitioners in Brazil [33]. Throughout the questionnaire some questions were adapted from a previous study investigating the mental health-related knowledge and practices of rural doctors in India [20].

The questionnaire was translated into the local language (Kannada) and was back translated into English to check for equivalence of meaning, and was subsequently piloted in a neighbouring area.

The vignettes for depression and psychosis were:

*Depression* - Meena is 30 years old and was fine until six months ago when she began to feel tired all the time. She says that she is sad and has lost interest in life. Even her children and family don’t make her feel happy. She cannot sleep, and she has lost the taste for food, which she used to love. She has also lost interest in cooking because she can’t concentrate. Sometimes she feels like jumping in the well to end her life.

*Psychosis -* Ram is 21 years old and is not married. He used to regularly help his father work on the farm, but for the last 10-15 days he has not been going to work. For the last 2-3 months he has been staying alone and aloof. He has not been bathing regularly and sometimes he becomes aggressive for no apparent reason, and once he even tried to hit his parents. He never used to behave in this way. On several occasions his father has found him talking to himself when nobody else was around. He has become suspicious of others and says that people are talking about him and that some people are keeping watch on him. For the last one week he has refused to eat food as he suspects his food is being poisoned by the neighbours. But his father refutes any truth in his suspicions.

### Analysis

Data from all the questionnaires was entered into a Microsoft Access database. This database was then uploaded into SPSS version 18.0 for analysis. Primary analysis involved using univariate statistics to describe the responses to the variables in the questionnaire. Percentages were calculated excluding any missing cases.

Secondary analysis involved the construction of an negative attitudes scale as follows: Participants had been presented with five statements that sought to assess the prevalence of negative attitudes and were asked whether they strongly agreed, agreed, were uncertain, disagreed, or strongly disagreed. A scale was then constructed, with each response to a negative attitude question given a score between one and five (5 = strongly agree, 4 = agree and so on). The scores for each question were then summed and each participant given a total score between 5 and 25, with 5 being the least negative. Independent-samples t-tests and Pearson’s r tests were performed to examine associations between endorsement of these negative attitudes and participant characteristics.

## Results

### Demographics and practice information

There were 13 of the 72 doctors who were unable to be contacted by the researcher due to being on leave or because clinics in very remote areas were sometimes unstaffed at the times the researcher was in the area. Among the 59 contactable participants, 78.0% (n = 46) consented to participate in the study. More than half of the participants were male (60.9%, n = 28) and the mean age of participants was 42 (range: 27 – 67). Almost all (93.5%, n = 43) participants were MBBS-trained, and just over half (58.7%, n = 27) were practising in the private sector while the remaining 41.3% (n = 19) were practising in either the government PHC clinics or the government Taluk hospital.

The mean total number of patients seen by participants in the week prior to completing the questionnaire was 260 (range 40–1000) and, on average, participants reported that 7 of their weekly patients presented with mental health problems (range: 0 – 50). If we take the average number of weekly patients presenting with mental health problems over the average number of total weekly patients then, on average, only 2.7% (7/260) of patients presented with mental health problems. General counselling, was the most common method (78.3%, n = 36) employed by the participants when treating patients with mental health problems, followed by specialist referral (67.4%, n = 31), prescription of medications (58.7%, n = 27), and other practices (10.9%, n = 5). Just over one fifth (21.7%, n = 10) reported that they either rarely or never prescribed anti-depressants.

### Training in mental health

Almost one third of the participants (28.0%, n = 13) had not received any training in caring for patients with mental health problems, including during their university study. Most participants had received their mental health training after (39.1%, n = 18) or during (37.0%, n = 17) their internship rather than during their university study (15.2%, n = 7).

Of the 33 (72.0%) participants who had received some type of mental health training, 19 (57.0%) reported that the training was one week or less in duration. All participants who had received training expressed that it was at least partly relevant to their practice. The most commonly identified areas of training received (see Table 1) were managing depression and anxiety (63.0%, n = 29), referring to a mental health professional (52.2%, n = 24), and promoting mental health in the community (50.0%, n = 23). The least commonly identified areas of training received were managing severe mental disorders (15.2%, n = 7), risk assessment (17.4%, n = 8), and undertaking a psychosocial needs assessment (19.6%, n = 9). The majority (85.0%, n = 39) of participants reported that they wanted to receive more training in how to provide mental health services.

**Table 1:** Receipt of mental health training in various topics (n = 46)

**Field**	**% (n) who had received training in this field**
Managing Depression and Anxiety	63.0 (29)
Referring to mental health professionals (e.g. psychiatrists)	54.3 (25)
Promoting mental health in the community	50.0 (23)
Counselling patients with Depression and Anxiety	47.8 (22)
Prescription of medications for mental health problems	41.3 (19)
Assessing the mental state of a patient	39.1 (18)
Counselling patients with problem drinking	32.6 (15)
Communication skills training	32.6 (15)
Managing patients who are problem drinkers	26.1 (12)
Managing patients who are violent	23.9 (11)
Managing patients who are suicidal	23.9 (11)
Psychosocial needs assessment	19.6 (9)
Risk Assessment	17.4 (8)
Managing severe mental disorders (e.g. psychoses)	15.2 (7)

### Knowledge of mental health, and self perceived competence in providing mental health care

The majority of participants (69.6%, n = 32) felt competent in providing mental health services to their patients, and most participants felt confident in recognising the signs and symptoms of; 1) depression and anxiety (78.3%, n = 36), and 2) psychosis (76.1%, n = 35). Participants were read the depression vignette and the psychosis vignette and asked to name the problem. Only ‘depression’, ‘schizophrenia’ or ‘psychosis’ were considered correct responses to the relevant vignettes. A majority (76.1%, n = 35) of participants correctly recognised the disorder in the depression vignette, and similarly, 76.1% (, n = 35) correctly recognised the disorder in the psychosis vignette. Just over half (58.7%, n = 27) of the participants correctly recognised the disorders in both vignettes.

Participants were also asked to name three signs and symptoms they believed to be common in patients with depression and schizophrenia/psychosis. Half (50.0%, n = 23) were able to correctly name three signs and symptoms of depression and, on average, participants correctly identified 2.4 signs and symptoms of depression out of a possible 3.0. Less than one third (28.3%, n = 13) were able to correctly name three signs and symptoms of schizophrenia/psychosis and, on average, participants correctly identified 2.0 signs and symptoms of schizophrenia/psychosis out of 3.0.

### Attitudes towards mental health

Participants were presented with a number of statements and asked to express their reaction to these statements from strongly agree to strongly disagree, with uncertain as an alternative middle option. These statements sought to elicit participant’s attitudes towards mental health service provision in the community, and the prevalence of negative attitudes towards mental health. These attitude statements are presented in Table 2, and responses are collapsed into agree, uncertain, or disagree.

**Table 2:** Responses to attitude statements (n = 46)

	**Agree**	**Uncertain**	**Disagree**
	**% (n)**	**% (n)**	**% (n)**
**Attitudes regarding mental health service provision**			
Medications usually produce a satisfactory result in patients with mental health problems	93.5 (43)	2.2 (1)	4.3 (2)
Mental health problems are common in the community.	87.0 (40)	6.5 (3)	6.5 (3)
The number of patients I see presenting with signs and symptoms of mental health problems is increasing.	82.6 (38)	6.5 (3)	10.9 (5)
The majority of mental health problems that I see originate from patients’ recent misfortunes	52.2 (24)	23.9 (11)	23.9 (11)
An underlying biochemical abnormality is at the basis of severe mental health problems	50.0 (23)	39.1 (18)	10.9 (5)
Medication is more beneficial than counselling for patients with mental health problems	43.5 (20)	15.2 (7)	41.3 (19)
Mental health problems can improve if nothing is done to treat them	19.6 (9)	0.0 (0)	80.4 (37)
**Negative attitudes statements**			
Mental Health problems are a sign of personal weakness	58.7 (27)	17.4 (8)	23.9 (11)
Working with patients with mental health problems is difficult	52.2 (24)	2.2 (1)	45.7 (21)
PHC doctors have little to offer patients with mental health problems	50.0 (23)	4.3 (2)	45.7 (21)
Counselling for mental health problems should be left to a specialist	34.8 (16)	8.7 (4)	56.5 (26)
Counselling tends to be unsuccessful in patients with mental health problems	17.4 (8)	17.4 (8)	65.2 (30)

The vast majority of participants agreed that mental health problems were common in the community (87.0%, n = 40), and that the number of patients they see with mental health problems is increasing (82.6%, n = 38). It was commonly believed that mental health problems need to be treated in order for them to improve (80.4%, n = 37). There was a commonly held belief that medications produce a satisfactory result for patients with mental health problems (93.5%, n = 43), although less than half (43.5%, n = 20) agreed that medications are more beneficial than counselling. There was no unanimously held view regarding whether mental health problems originate from recent misfortunes (52.2%, n = 24) or a biochemical abnormality (50.0%, n = 23).

There was a substantial level of endorsement for several statements that could be construed as negative or reflecting negative attitudes regarding both people with mental health problems and participant’s perception of their capacity to provide care for such people. Approximately three quarters either agreed (58.7%, n = 27) or were uncertain (17.4%, n = 8) when presented with the statement ‘mental health problems are a sign of personal weakness’. Approximately half agreed that it is difficult to work with mental health patients (52.2%, n = 24), and that PHC doctors have little to offer these patients (50%, n = 23). A minority of participants believed that counselling should be left to specialists (34.8%, n = 16) and that counselling tends to be unsuccessful (17.4%, n = 8).

The negative attitudes score, constructed from these five statements, showed satisfactory internal consistency (Cronbach’s alpha = 0.70). The overall mean score was 14.6 out of 25.0. Agreement with these negative attitudes statements was higher among; 1) doctors practising in private clinics t(26) = 2.39, p = .024, 2) doctors who had not received any mental health training t(36) = 2.61, p = .013, and 3) doctors who did not feel competent in providing mental health services t(43) = 3.16, p = 0.003. There was no statistically significant association with age r(44) = .280 p = 0.059 or the number of patients seen per week r(44) = .130 p = 0.391.

## Discussion

There is a wide gap between the mental health needs of the community and the available mental health services in low and middle income countries [3]. Integration of mental health into PHC is one solution to the treatment gap, and requires task shifting from mental health specialists to a range of non-specialist health workers including doctors providing PHC [16]. Few studies have examined the mental health knowledge, attitudes and practices of primary care doctors in India. This study makes a contribution to the global mental health literature by conducting a knowledge and attitudes survey among public and private sector primary care doctors in a rural district of India.

The PHC doctors participating in this study were operating in a busy practice context, with an estimated average of 260 patients per week. The very limited amount of time the doctors are able to spend with each patient creates an obvious challenge for providing mental health care, and this is a larger issue to be faced by programs aiming to increase integration of mental health into PHC. Research has indicated that longer consultations are associated with greater detection of psychological problems [34], and that if doctors do not have the necessary brief screening questions in mind they may miss the opportunity to detect and treat a high proportion of mental disorders [35].

On average, participants reported that just 2.7% of their patients presented with mental health problems, in contrast to the estimated national prevalence of 6% [3]. This discrepancy could in part be explained by a lack of diagnostic capacity among the participants in addition to a lack of awareness of mental health in the community. Patients may be unlikely to present/self-identify as having a problem with their mental health and doctors may not be sufficiently attuned to exploring the potential for mental health problems among patients presenting with physical manifestations of distress. Another explanation could be that the participants have simply under-estimated the number of their patients that they have determined to have mental health problems.

Effective, evidence-based and targeted training in mental health for PHC practitioners is critical to ensure effective integration of mental health into PHC settings. The findings from our study indicate that, in this one rural district in the south of India, almost one third of PHC doctors had not received any training at all in mental health. The duration of mental health training that had been received was typically one week or less, and very few had received any training in mental health during their graduate study. Given the diagnostic and management skills required to adequately provide mental health care in PHC settings it is unlikely that one-off short-term training with minimal follow-up and supervision is going to be sufficient. Our findings are comparable to those from a similar study among allopathic, homeopathic, and ayurvedic medical practitioners in the city of Ludhiana (Punjab) which found 27.1% had received no training at all in dealing with patients suffering from mental health problems, and just 30.1% had received training during their graduate study [20]. Encouragingly, the vast majority of participants in our study reported that they wanted to receive more training in the provision of mental health services within the PHC setting.

There was a high degree of self-perceived competence among the PHC doctors in our study. Despite the limited access to training in mental health, the majority of participants felt confident in providing mental health care to their patients and in recognising the signs and symptoms of depression and anxiety, and psychosis.

The majority of participants were able to correctly recognise depression (76.1%) and psychosis (76.1%) in vignettes. These figures are substantially higher than pre-test results from a training evaluation study using the same vignettes among community health workers in the same locality (depression - 22.9%; psychosis - 8.6%) [31], indicating that the PHC doctors in our study had a greater capacity to recognise mental health disorders.

However, only half of the participants in our study were able to correctly name three signs and symptoms of depression and this dropped to less than one-third for psychosis. These results suggest that, whilst the majority of the PHC doctors had sufficient general knowledge of mental health to recognise disorders in a vignette, a substantial proportion did not have sufficient depth of knowledge to be able to name three signs and symptoms of depression and psychosis. This is in contrast to the high degree of self-perceived competence among the participants regarding their capacity to recognise such signs and symptoms and treat patients appropriately.

There was a high level of interest in mental health among the participants. Almost all of the PHC doctors believed that mental health problems were common in the community. There was no unanimously held view regarding whether medications are more beneficial than counselling, nor whether mental health problems originate from patient’s recent misfortunes or a biochemical abnormality. Medications were widely endorsed by participants which is not surprising given that the practice of psychiatry in India is strongly influence by psychopharmacology [36].

Negative and stigmatising attitudes were commonly endorsed by the participants. For instance, it was commonly thought that mental health problems were a sign of personal weakness (58.7% agree, 17.4% uncertain); this attitude was also endorsed by community health workers in a study in the same locality [31]. Furthermore, many participants appeared to see only a limited role for PHC doctors with regard to providing mental health care. Negative attitudes and the related concept of stigma have a substantial impact on the care of people with mental health problems. Stigma in the context of mental disorders is well documented [37,38] and is linked to discrimination [39,40], under-use of mental health services, delay in receipt of treatment, and an impeded recovery process [41-44]. Previous studies have found that training that focuses on evidence for the effectiveness of treatment [41,45] and provides contact with people with mental health problems (e.g. face to face encounters with people who have recovered) [46-48] has the potential to reduce negative attitudes.

Negative attitudes were more commonly endorsed by 1) doctors practicing in private clinics, 2) doctors who had not received any mental health training, and 3) doctors who did not feel competent in providing mental health services. It is important that any mental health training and support initiatives are extended to doctors operating in private clinics in addition to their government counterparts.

### Limitations

This study was relatively small in scope and only collected data from one sub-district. The results cannot be generalised outside of this district, although the similarities with findings from previous studies suggest there are some common themes. A small number of doctors were unable to be contacted due to logistical difficulties associated with being in isolated areas, and some doctors declined to participate. It is possible that doctors who declined participation or were practising in more isolated areas may have different characteristics and mental health skills to the ones who participated in this study.

The study could have been improved by the inclusion of questions related to the management of suicide, particularly given suicide is a major public health issue in India [49]. Furthermore, patients’ experiences of mental health care as provided by their primary care doctor needs to be explored.

## Conclusion

The strategy of integrating mental health into PHC settings has great value. The necessary task shifting that flows from this strategy will require systematic planning and the implementation of evidence-based up-skilling programs for a range of health personnel. The doctors in this study were operating in a very busy PHC practice context, and their capacity to provide mental health care had clear limitations. Negative and stigmatising attitudes related to mental health were commonly endorsed although there was a high level of interest in knowing more about mental health. There was a surface level of knowledge among the doctors about the signs and symptoms of mental health, however, the doctors commonly lacked a sufficient depth of knowledge to name three signs and symptoms of depression and psychosis. The doctors desired greater training and support to enable them to provide mental health care in their PHC settings. Continuing mental health education for PHC doctors in India represents a priority, particularly in rural areas where there are few if any psychiatrists. More evidence is needed to identify the best models of up-skilling doctors and other non-specialist health workers so that they are able to competently provide basic and effective mental health care.

## Competing interests

The authors declare that they have no competing interests.

## Authors' contributions

GA and JC held primary academic responsibility for the conduct of the study, and SR provided in-country supervision and direction. JC and GA developed the data collection tool and methods. JC undertook the data collection in Doddaballapur. JC and GA undertook the data analysis and drafted the manuscript. AN provided expertise regarding the context of primary health care in Doddaballapur and supported the recruitment of participants. All authors contributed to the interpretation of the results, and have read and approved the manuscript.
